# Peptide-Based Turn-On Fluorescent Probes for Highly Specific Detection of Survivin Protein in the Cancer Cells

**DOI:** 10.1021/cbmi.4c00017

**Published:** 2024-05-02

**Authors:** Takeshi Fuchigami, Tomoe Nakayama, Yusuke Miyanari, Iori Nozaki, Natsumi Ishikawa, Ayako Tagawa, Sakura Yoshida, Masayuki Munekane, Morio Nakayama, Kazuma Ogawa

**Affiliations:** † Laboratory of Clinical Analytical Sciences, Graduate School of Medical Sciences, Kanazawa University, Kakuma-machi, Kanazawa, Ishikawa 920-1192, Japan; ‡ Department of Hygienic Chemistry, Graduate School of Biomedical Sciences, Nagasaki University, 1-14 Bunkyo-machi, Nagasaki 852-8521, Japan; § Institute of Nano Life Science, Kanazawa University, Kanazawa, Ishikawa 920-1192, Japan; ∥ Institute for Frontier Science Initiative, Kanazawa University, Kakuma-machi, Kanazawa 920-1192;, Japan

**Keywords:** Survivin, Borealin, Peptides, Responsive fluorescence imaging, Cancer imaging

## Abstract

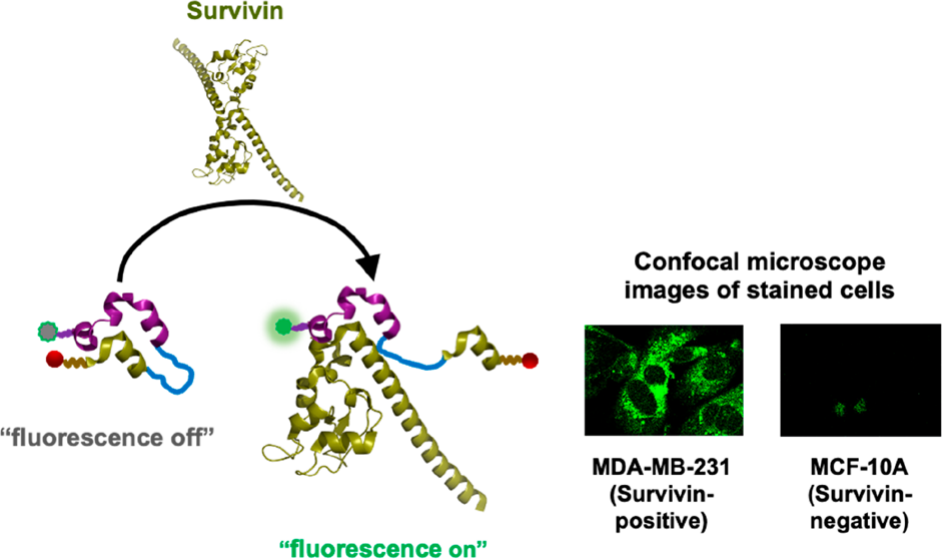

Survivin is highly expressed in most human cancers, making it a promising target for cancer diagnosis and treatment. In this study, we developed peptide probes consisting of Bor_65–75_, a high-affinity survivin-binding peptide, and a survivin protein segment using peptide linkers as survivin-sensitive fluorescent probes (SSFPs). All conjugates were attached to 5(6)-carboxyfluorescein (FAM) at the *C*-terminal as a fluorophore and to 4((4(dimethylamino)phenyl)azo)benzoic acid (DABCYL) at the *N*-terminal as a quencher. Fluorescence (or Förster) resonance energy transfer (FRET) quenching via intramolecular binding of Bor_65–75_ with survivin protein segment could be diminished by the approach of survivin to SSFPs, which dissociate Bor_65–75_ from SSPF and increased the distance between FAM and DABCYL. A binding assay using recombinant human survivin protein (rSurvivin) demonstrated moderate to high affinity of SSFPs for survivin (dissociation constants (*K*_d_) = 121–1740 nM). Although the SSFPs (0.5 μM) had almost no fluorescence under baseline conditions, a dose-dependent increase in fluorescence intensity was observed in the presence of rSurvivin (0.1–2.0 μM). In particular, the proline-rich SSFP (SSFP5) showed the highest (2.7-fold) fluorescence induction at 2.0 μM survivin compared to the signals in the absence of survivin. Confocal fluorescence imaging demonstrated that SSFP5 exhibited clear fluorescence signals in survivin-positive MDA-MB-231 cells, whereas no marked fluorescence signals were observed in survivin-negative MCF-10A cells. Collectively, these results suggest that SSFPs can be used as survivin-specific FRET imaging probes.

## Introduction

Survivin is an inhibitor of apoptosis protein (IAP), consisting of 142 amino acids, and was discovered by Ambrosini et al. in 1997.^1,2^ This protein is expressed in most human cancer tissues, but is absent in normal differentiated tissues.^3,4^ Survivin serves two important functions: inhibition of apoptosis and regulation of mitosis.^5,6^ Aberrant expression of survivin is closely related to poor prognosis in cancer.^7^ Owing to the restricted expression and importance for cancer survival and progression, survivin is a potential target for cancer diagnosis and therapy.^8,9^ Thus, specific imaging probes for survivin may lead to further elucidation of cancer biology involving survivin and cancer-specific diagnosis.

**Figure 1:**
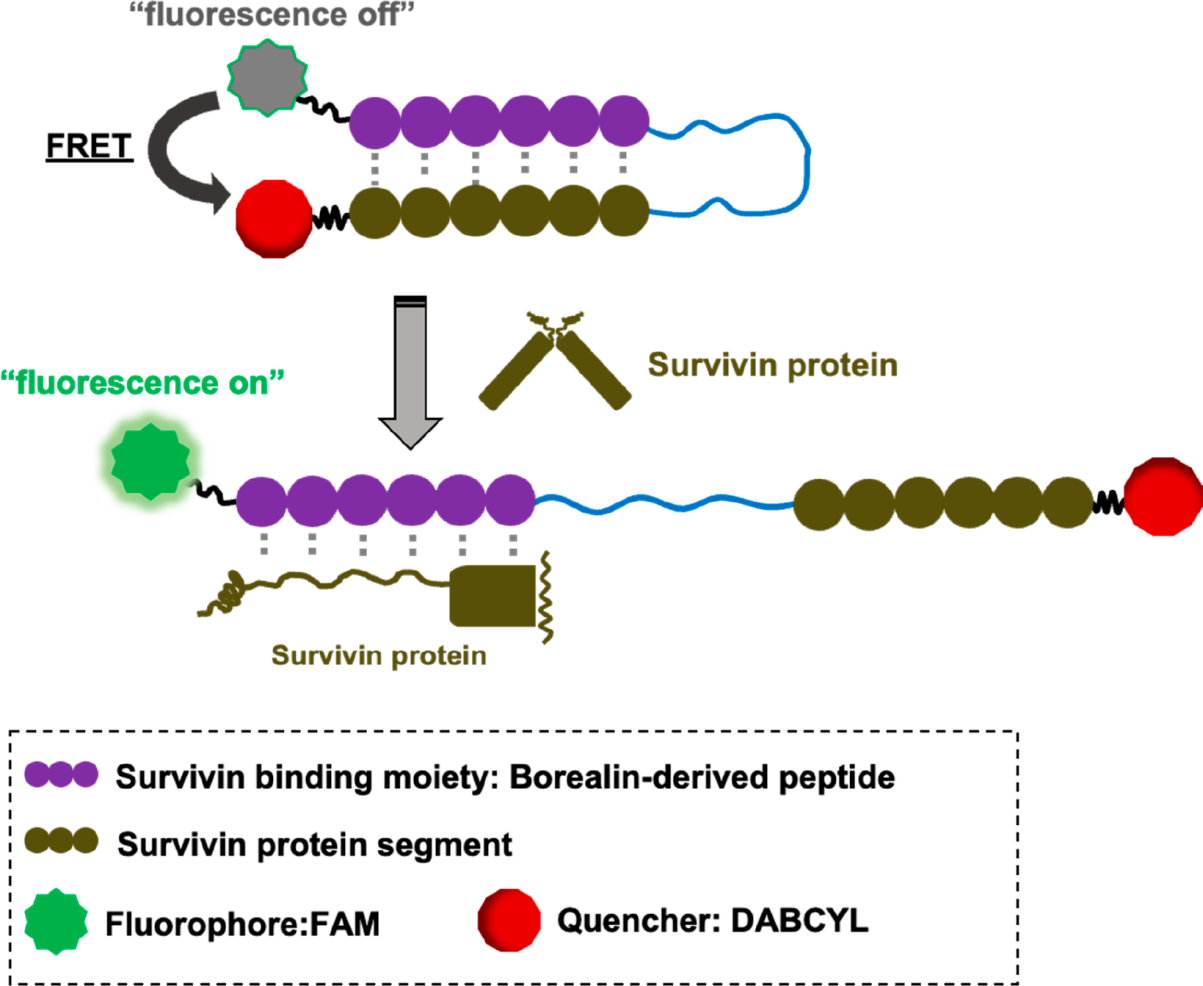
Strategy of activatable fluorescent probes that specifically recognize survivin. Each designed survivin-sensitive fluorescent probe (SSFP) possesses a high-affinity survivin-targeting peptide and a survivin protein segment corresponding to the survivin-targeting peptide-binding site, and then a fluorescent and a quencher molecule are placed close together. Fluorescence signals of fluorophore 5(6)-carboxyfluorescein (FAM) are quenched by the intramolecular quencher 4((4(dimethylamino)phenyl)azo) benzoic acid (DABCYL) owing to the interaction between survivin-targeting peptide and survivin protein segment. The binding of the molecular probes to survivin is expected to enhance the fluorescence of the donor fluorophore.

Recently, fluorescence-based nanosensors for live imaging of survivin mRNA expression in mammalian cells were developed.^10^ Evaluation of survivin mRNA changes may be useful in elucidating the relationship between survivin transcriptional activity and cancer progression and in predicting prognosis. Generally, protein expression does not align with mRNA expression because of various translational and subsequent processing factors. In addition, specific detection of survivin protein expression and dynamics is expected to provide insights into cancer progression through the interaction of survivin with other proteins as well as drug discovery tools. Therefore, it is desirable to develop imaging probes that can specifically detect survivin protein in cancerous tissues. However, there are no survivin-specific imaging probes that can be imaged using nuclear medicine, fluorescence, ultrasound, or other modalities, and their development is urgently required. Numerous small-molecule compounds targeting survivin have been reported, most of which function by either inhibiting survivin expression or interfering with signaling pathways.^11−14^ We recently developed several agents that directly target survivin based on small molecules^15,16^ and peptide derivatives.^17,18^ Among them, Bor_65–75_ (LREMNWLDYFA-NH_2_), an 11-amino acid peptide derived from Borealin (Bor peptide), exhibits the highest binding affinity (dissociation constants (*K*_d_) = 49.6 ± 11 nM) for survivin. However, fluorescence-labeled Bor_65–75_ still shows considerable nonspecific binding to survivin-negative regions in cancer cells.^18^ Therefore, further optimization of the imaging probe structure is necessary for the specific detection of survivin. To achieve this goal, we designed survivin-activatable fluorescence imaging probes consisting of a high-affinity survivin-targeting peptide and a survivin protein segment corresponding to the survivin-targeting peptide-binding site and then placed a fluorescent molecule and a quencher molecule close together (Figure 1). Such imaging probes are expected to exhibit fluorescence quenching based on the principle of fluorescence (or Förster) resonance energy transfer (FRET) when the fluorescent and quencher molecules are in close proximity in the folded conformation, where the survivin-binding moiety and survivin protein segment are bound by intramolecular hydrogen bonds or hydrophobic interactions. Binding of the Bor_65–75_ region of the FRET probes to the survivin protein is expected to lead to a divergence of the survivin protein segment within the molecule. This is expected to result in a distance between the fluorescent dye and the quencher molecule, causing fluorescence emission. Therefore, we named these FRET probes survivin-sensitive fluorescent probes (SSFPs). In this study, we synthesized several SSFPs with different linkers and survivin target peptide sequences and examined whether these FRET probes are useful for specific detection of survivin protein in the biological samples.

## Experimental Section

### General Information

All reagents were commercial products and were used without further purification, unless otherwise indicated. *N*-9-Fluorenylmethoxycarbonyl (Fmoc)-NH SAL resin, Fmoc amino acids, 1-[bis(dimethylamino)methylene]-1H-benzotriazolium 3-oxide hexafluorophosphate (HBTU), 1-[bis(dimethylamino)methylene]-1*H*-1,2,3-triazolo[4,5-*b*]pyridinium 3-oxide hexafluorophosphate (HATU), and 1-hydroxybenzotriazole (HOBt) were purchased from Watanabe Chemical Industries Co., Ltd. (Hiroshima, Japan). 3*H*-[1,2,3]Triazolo[4,5-*b*]pyridin-3-ol (HOAt) was obtained from Toronto Research Chemicals (Toronto, Canada). 5(6)-Carboxyfluorescein diacetate succinimidyl ester (5(6)-CFSE) was synthesized according to the literature.^19^ Mass spectra were obtained with matrix-assisted laser desorption/ionization time-of-flight mass spectrometry (MALDI-TOF-MS) using Ultraflex MALDI TOF/TOF MS (Bruker Daltonics, Bremen, Germany). HPLC analysis was performed by a Shimadzu HPLC system (LC-10AT pump with SPD-10A UV detector, λ = 254 nm).

### Peptide Synthesis

All peptides were synthesized through a stepwise solid-phase method by Fmoc chemistry with Fmoc-amino acid on 100 mg of Fmoc-NH-SAL resin (0.55 mmol amine/g resin), utilizing the Biotage Initiator + Alstra system (Biotage Japan, Tokyo, Japan). The resin was initially immersed in *N*,*N*-dimethylformamidine (DMF) for 18 h. Subsequently, the Fmoc group was deprotected with 20% (v/v) piperidine in DMF. Fmoc-amino acid (0.165 mmol) and a condensing agent cocktail (0.165 mmol) of HBTU: HOBt = 1:1 (for SSFP1, SSFP2, SSFP3, and SSFP5) or HATU: HOAt = 1:1 (for SSFP4), along with *N*,*N*-diisopropylethylamine (DIPEA) (0.33 mmol), in DMF were added. The mixture was shaken at 75 °C for 15 min and then washed with DMF. The peptide chains were elongated by repeating this procedure. 4((4(Dimethylamino)phenyl)azo) benzoic acid (DABCYL) (3 equiv), HBTU (3 equiv), HOBt (3 equiv), and DIPEA (6 equiv) were added and the mixture was shaken overnight under shielded light to yield *N*-DABCYL-labeled peptides. The 1-(4,4-dimethyl-2,6-dioxocyclohexylidene)-3-methylbutyl (ivDde) protecting group of the Lys residue was cleaved using 10% hydrazine monohydrate in DMF. Then, 5(6)-CFSE (3 equiv) and DIPEA (6 equiv) were added and shaken overnight under shielded light to yield *C*-5(6)-carboxyfluorescein (FAM)-labeled peptides. Upon completion of labeling, the peptide was cleaved from the resin using trifluoroacetic acid (TFA):H_2_O:triisopropyl silane (TIS):1,2-ethanedithiol (EDT) (94:2.5:1:2.5 v/v/v/v) for 90 min with shaking at room temperature. After separating the peptide from the resin, the filtrate was precipitated with chilled diethyl ether. The precipitate was centrifuged at 2,500 relative centrifugal force (rcf) for 5 min, washed with diethyl ether 3 × , and centrifuged in between each washing step. The crude products were purified by HPLC on a Cosmosil C_18_ column (Nacalai Tesque, 5C_18_-AR-II, 10 × 250 mm) using a water (0.1% TFA)–acetonitrile (0.1% TFA) gradient at a flow rate of 2.0 mL/min. The purified products were analyzed by HPLC on a Cosmosil C_18_ column (Nacalai Tesque, 5C_18_-AR-II, 4.6 × 150 mm) using a water (0.1% TFA)–acetonitrile (0.1% TFA) gradient at a flow rate of 1.0 mL/min. Each peptide was analyzed by MALDI-TOF-MS.

### Cell Cultures

MDA-MB-231 cells (human breast cancer cells) and MCF-10A cells (human breast nontumorigenic epithelial cells) were obtained from the American Type Culture Collection (Manassas, VA, USA). MIA PaCa-2 cells (human pancreatic cancer cells) were purchased from the RIKEN BioResource Research Center (Tsukuba, Japan). MDA-MB-231 cells and MIA PaCa-2 cells were cultured in Dulbecco’s Modified Eagle’s Medium (DMEM) (low glucose) supplemented with 10% fetal bovine serum (FBS). MCF-10A cells were grown in DMEM/F-12 supplemented with 5% horse serum, 20 ng/mL epidermal growth factor (EGF), 10 μg/mL insulin, and 0.5 μg/mL hydrocortisone. All media were supplemented with 100 IU/mL penicillin and 100 μg/mL streptomycin. Cells were maintained in a humidified 5% CO_2_ incubator at 37 °C.

### Saturation Binding Assay of SSFPs to recombinant human survivin protein (rSurvivin)

The rSurvivin was expressed and purified as described in the previous report.^14^ The binding assay of SSFPs to rSurvivin was performed using the quartz crystal microbalance (QCM) system (Affinix Q, Initium Inc., Tokyo, Japan) according to our previous reports.^17,18^ rSurvivin (40 μg/mL) was immobilized on the gold electrode of the sensor chip through amide bonds. The sensor chip was placed onto the QCM apparatus and immersed in a buffer. Subsequently, an 8 μL aliquot of each SSFP (10–1300 nM) was sequentially injected into the cuvette, and frequency changes were monitored over time. The kinetic analysis was carried out using AQUA ver. 1.3 software (Initium Inc.).

### Measurement of Fluorescence Spectrum and Intensity of SSFPs

SSFP solutions of 0.5 μM mixed with 0.1–2.0 μM rSurvivin or human serum albumin (HSA) were prepared in 96-well microplates. A multimode reader (Cytation3, Agilent Technologies, Santa Clara, CA, USA) was used to measure fluorescence intensity of each mixture in the microplate. FAM was excited at 483 nm, and the fluorescence spectrum was detected at 510–600 nm. The fluorescence spectra and intensities of FAM as a positive control and DABCYL as a negative control were measured using the same method. The fluorescence intensity was measured using a multimode plate reader, and the FRET efficiency was calculated using the following equation:
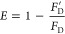
where *F*’_D_ represents the fluorescence intensity of the donor in the presence of an acceptor and F_D_ represents the fluorescence intensity of the donor in the absence of an acceptor.

### Confocal Fluorescence Imaging of Cells with SSFPs

Cultured MDA-MB-231 and MCF-10A cells were fixed with formaldehyde and permeabilized with Triton X-100. The cells were then incubated with SSFPs (5 μM) for 1 h and later washed with PBS. Immunofluorescence staining was performed using antisurvivin primary antibody (D-8) (Santa Cruz Biotechnology Inc., CA, USA) and Alexa Fluor 633 goat antimouse IgG(H+L) (Thermo Fisher Scientific Inc., Waltham, MA, USA) secondary antibody. Fluorescence images were captured by a confocal laser scanning microscope (LSM710, Carl Zeiss, Germany; excitation λ = 488 nm, emission λ = 494–601 nm for FITC, excitation λ = 633 nm, emission λ = 639–758 nm for survivin).

### Statistical Analysis

Statistical significance was determined using the one-way analysis of variance (ANOVA) for comparison of more than two means, followed by post hoc tests using Turkey’s correction for the evaluation of fluorescence intensity of SSFPs in the presence or absence of proteins (Figure 4). A value of *P* < 0.05 was considered statistically significant.

## Results and Discussion

### Design and Synthesis of SSFPs

**Figure 2:**
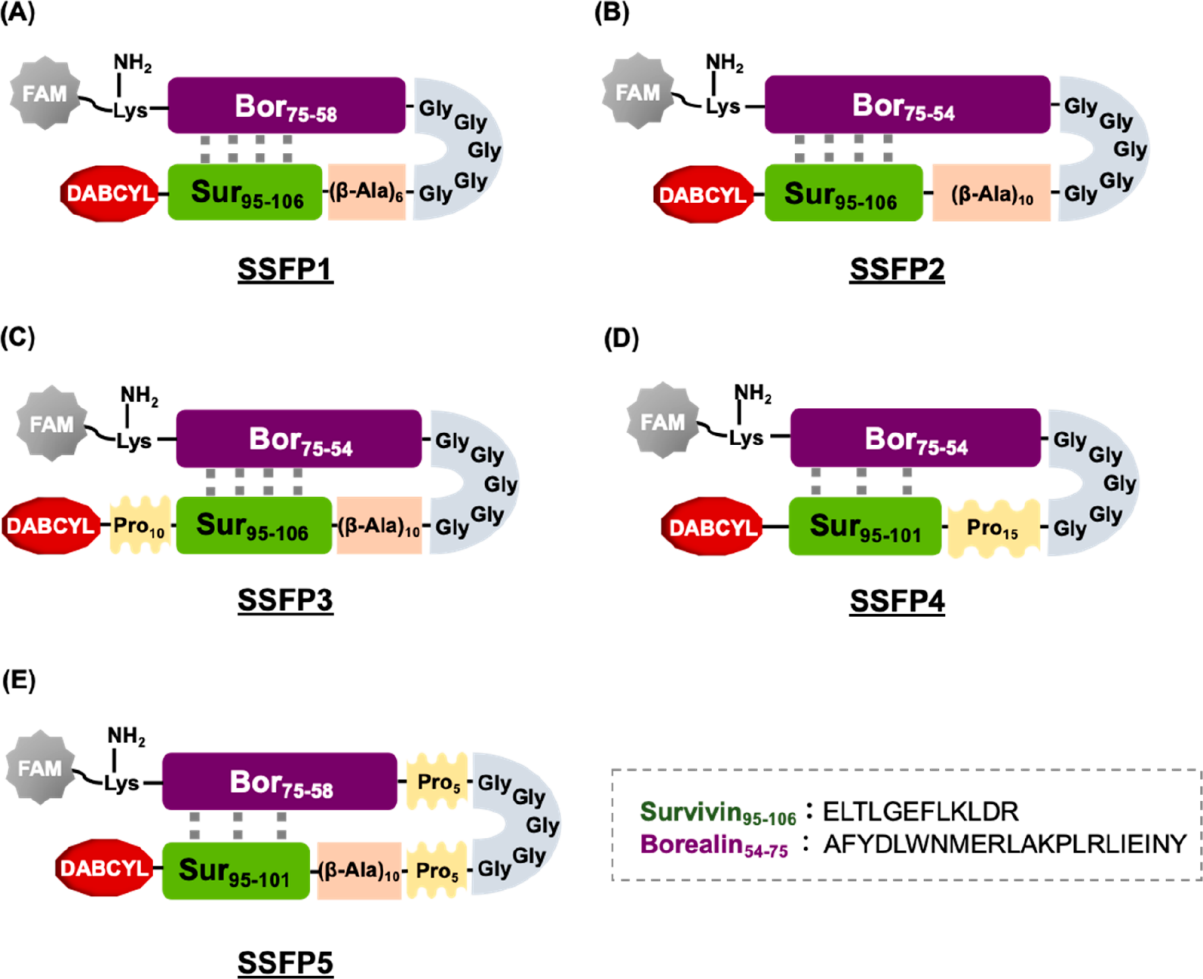
Molecular features of SSFP1 (A), SSFP2 (B), SSFP3 (C), SSFP4 (D), and SSFP5 (E) as survivin-sensitive fluorescent probes (SSFPs).

To facilitate FRET quenching, the emission spectrum of the donor fluorescent dye and the absorption spectrum of the acceptor quenching dye must align to be approximately 3–8 nm (30–80 Å) apart.^20,21^ Given that the size of a single amino acid is approximately 3 Å, FRET is deemed to be notably weak when the linear distance between the two molecules exceeds 27 residues in the context of peptides. In our previous studies on the development of survivin-targeting peptides, Bor_65–75_ showed the highest binding affinity to rSurvivin, with a *K*_d_ value of 49.6 nM.^18^ Therefore, we selected a peptide sequence containing the 95–110th amino acid residue of survivin protein segment, the region of direct binding interaction between Bor_65–75_ and survivin, linked to Bor_65–75_ via a linker to form a fusion peptide. FRET probes containing a pair of fluorophore FAM and DABCYL were reported to detect enzymes in the biological specimens.^22,23^ Using these FRET systems, we designed SSFPs that attached FAM to the *C*-terminal lysine side chain near the Bor_65–75_ sequence of the peptide as a fluorescent molecule and attached DABCYL to the *N*-terminus of the survivin protein segment as a quencher molecule. Concerning the molecular design of the linkers, we opted for flexible glycine and β-alanine linkers^24^ so that the molecular probes would readily adopt a folded conformation. We designed SSFP1 (Figure 2A) with FAM bound to the *C*-terminal Lys side chain with the Bor_75–58_ sequence nearby and DABCYL bound to the *N*-terminal side with Sur_95–105_ in the vicinity. SSFP2 (Figure 2B) was designed with a longer Bor peptide sequence than SSFP1 to assess whether this design would improve binding to survivin and reduce FRET quenching. Polyprolines have been used as linkers for FRET-based molecular probes because they have a rigid structure and remain constant in length and direction.^21^ Therefore, the incorporation of rigid polyproline linkers within the imaging probe is expected to result in greater separation between the fluorescent and quencher molecules when survivin binds, resulting in stronger fluorescence emission. For this reason, we designed SSFP3 (Figure 2C) with a polyproline linker incorporated on the *N*-terminal side, and SSFP4 (Figure 2D) and SSFP5 (Figure 2E) with polyproline linkers on one or both sides of the loop structure.

**Scheme 1:**
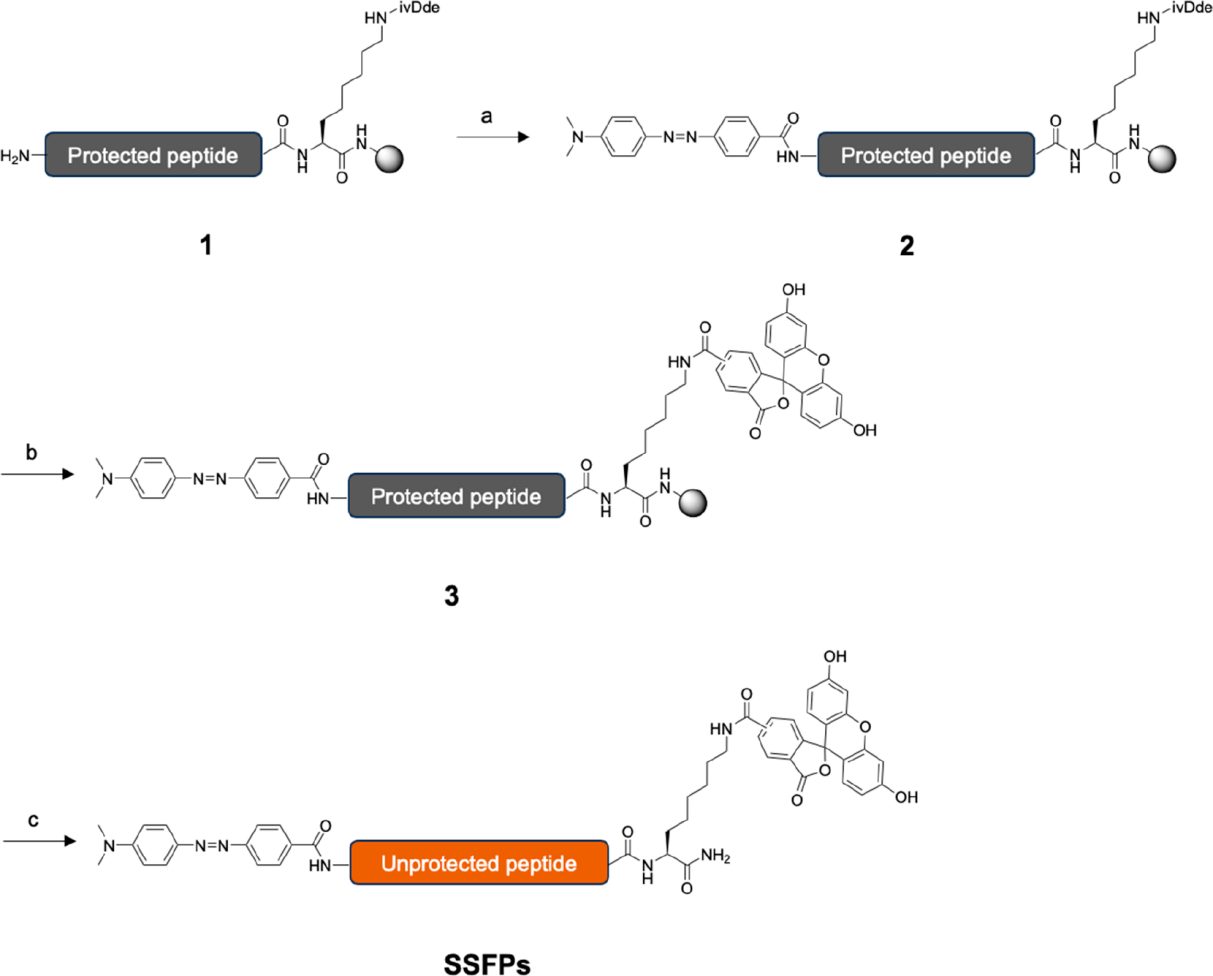
Synthesis of SSFPs Reagents and conditions: (a) DABCYL, HBTU, HOBt, DIPEA, rt, 12 h, (b) (1) hydrazine monohydrate, DMF rt, 12 h, (2) 5(6)-CFSE, DIPEA rt, 12 h, (c) TFA, water, TIS, EDT, rt, 90 min.

The SSFPs were synthesized using the Fmoc solid-phase synthesis method (Scheme 1). Rink Amide resin was first coupled with Fmoc-l-Lys(ivDde)–OH, and each peptide sequence was then elongated with Fmoc-amino acids using HBTU and HOBt as coupling agents to yield protected peptide **1**. In the synthesis of SSFP4, which has a long polyproline linker, HATU, and HOAt were used as coupling agents to prevent low chemical yields. DABCYL was coupled to the *N*-terminal amine group of **1** using HBTU and HOBt under basic conditions to produce DABCYL-conjugated peptide **2**. After deprotection of the ivDde group in *C*-terminal l-lysine with hydrazine, each peptide was coupled with 5(6)-CFSE to yield the DABCYL- and FAM-conjugated peptide **3**. Finally, the resin and other protecting groups were cleaved to produce the SSFPs. Crude peptides were purified using reverse-phase HPLC and identified as target peptides by MALDI-TOF-MS (Table S1). Analytical HPLC indicated that purified SSFPs have >95% purity (Figure S1).

### Binding Affinities of SSFPs for rSurvivin

**Figure 3:**
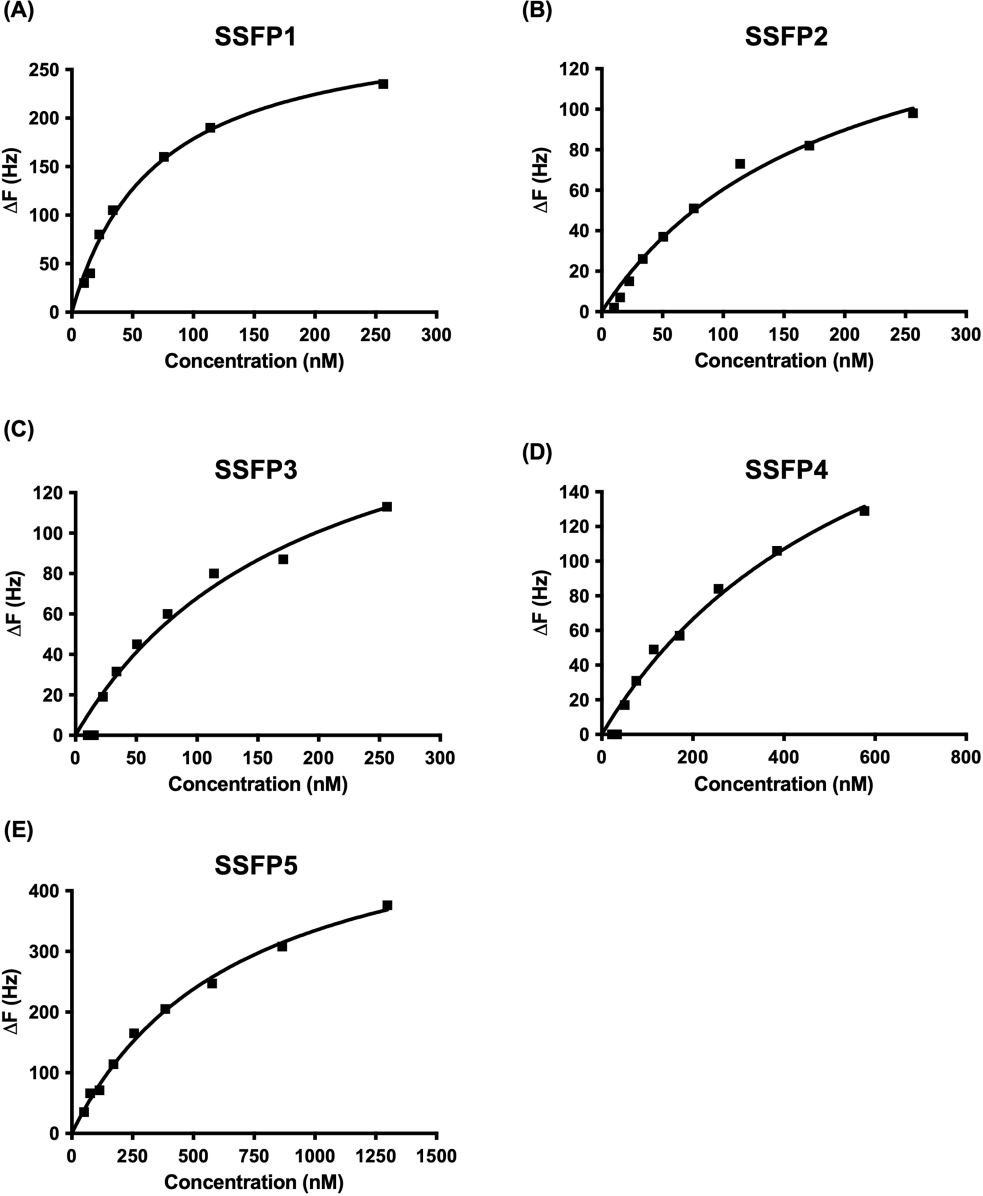
Representative binding saturation curves of SSFP1 (A), SSFP2 (B), SSFP3 (C), SSFP4 (D), and SSFP5 (E) with recombinant human survivin (rSurvivin). Δ*F* values (Hz) represent the frequency change of electrodes.

The binding affinities of SSFPs for rSurvivin were assessed by QCM, in which the *K*_d_ values were determined by immobilizing rSurvivin on a QCM plate and plotting the change in frequency caused by compounds binding to rSurvivin.^17^ These SSFPs showed saturation and fitted well with the one binding site model (Figure 3A–E). The *K*_d_ values of SSFPs for rSurvivin were evaluated that ranged from 203 to 1740 nM (Table 1), suggesting that all SSFPs have affinity for rSurvivin. When compared to the parent compound Bor_65–75_ (*K*_d_ = 49.6 ± 10.8 nM), the binding affinities of all SSFPs decreased. The *K*_d_ values of SSFPs tended to increase with increasing molecular weight, probably due to a decrease in the proportion of the overall peptide amino acid region that exhibits binding to survivin. Therefore, the usefulness of SSFPs as survivin imaging probes may not be determined solely by their affinity for the rSurvivin.

**Table 1:** Dissociation Constant (*K*_d_) Values of SSFPs for Survivin Determined by QCM Assay^a^

SSFPs	*K*_d_ (nM)
SSFP1	203 ± 65.1
SSFP2	257 ± 53.7
SSFP3	358 ± 68.1
SSFP4	1197 ± 315
SSFP5	1740 ± 413

a Values are mean ± standard error of the mean (SEM) for three to six independent measurements.

### Evaluation of SSFPs as FRET Probes

**Figure 4:**
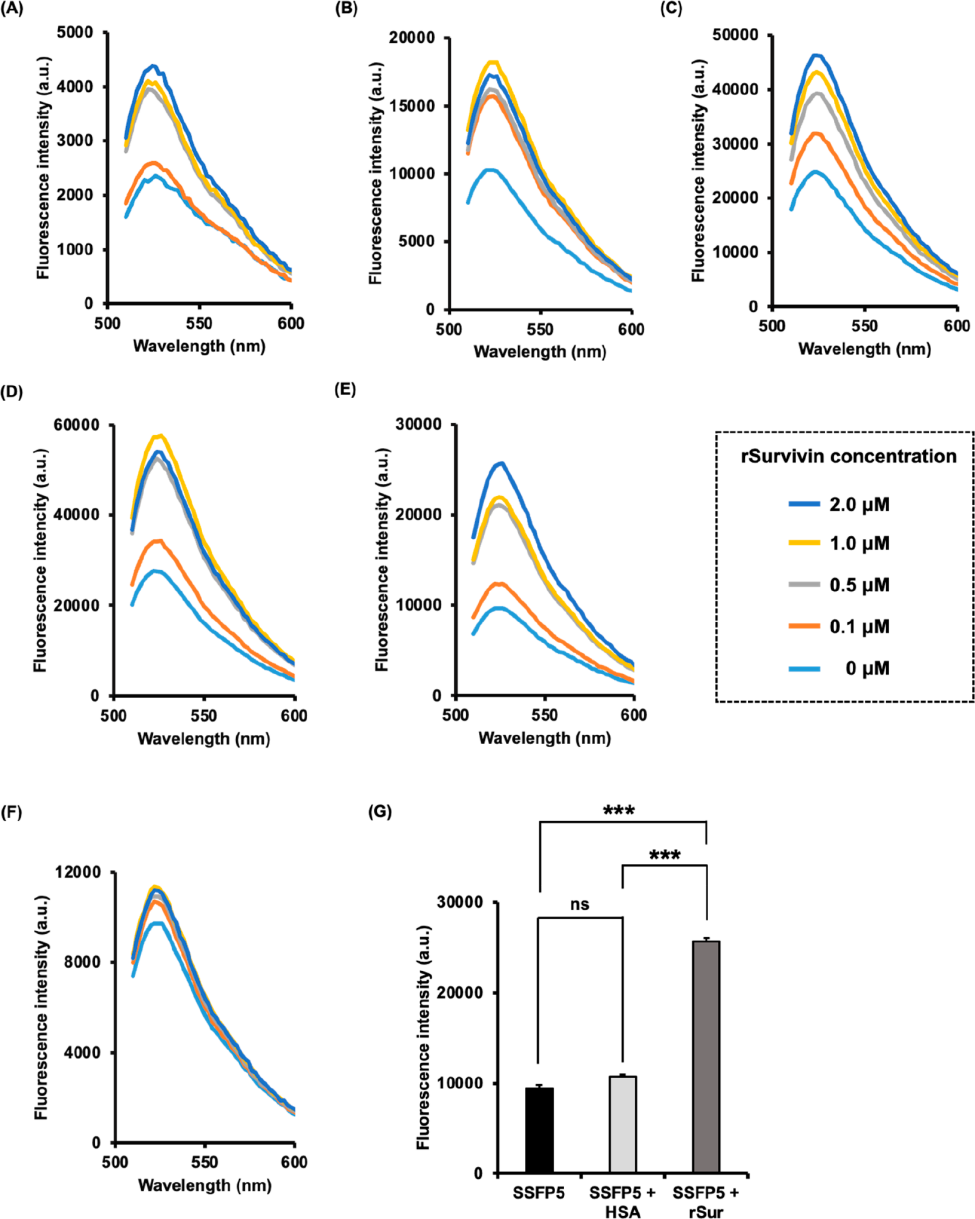
Fluorescence emission spectra of SSFP1(A), SSFP2 (B), SSFP3 (C), SSFP4 (D), and SSFP5 (E) in the presence of rSurvivin. Fluorescence emission spectra of SSFP5 in the presence of human serum albumin (HSA) (F). Fluorescence spectra of SSFP1–SSFP5; ex: 483 nm, em: 510–600 nm. Fluorescence intensity (ex = 483 nm, em = 525 nm) of SSFP5 (0.5 μM) in the absence or presence of HSA or rSurvivin (rSur) at 2.0 μM. Values are mean ± SEM (*n* = 3) (G). ***P < 0.001 (ANOVA, Tukey *t* test).

To confirm whether SSFPs underwent FRET quenching, the fluorescence intensities of 0.5 μM SSFPs, 0.5 μM DABCYL, and 0.5 μM FAM were measured. The results show that the fluorescence intensity of all SSFPs at 525 nm, the maximum fluorescence wavelength of FAM, was more than 25 times weaker than that of the donor FAM. The FRET quenching efficiency of the SSFPs exceeded 90%, indicating a strong FRET quenching (Table S2). Next, fluorescence measurements were conducted to assess whether the binding of SSFPs to rSurvivin induced a change in the fluorescence intensity resulting from FRET quenching. When 0.5 μM SSFPs were mixed with varying concentrations of rSurvivin ranging from 0 μM to 2.0 μM, the fluorescence intensity was measured, revealing an increase in the fluorescence intensity of SSFPs with higher amounts of rSurvivin (Figure 4). The fluorescence intensity of SSFP1, SSFP2, SSFP3, SSFP4, and SSFP5 increased by 1.85-, 1.79-, 1.88-, 2.15-, and 2.66-fold, respectively, when mixed with 2.0 μM rSurvivin compared to the mixture with no rSurvivin (Figure 4A–4E). SSFP5 exhibited the highest increase in fluorescence intensity upon binding to rSurvivin. In contrast, SSFP5 showed almost no change in fluorescence intensity (approximately 1.16-fold increase) when mixed with 2 μM HSA (Figure 4F). Furthermore, the quantified fluorescence intensity of SSFP5 (5 μM) in the presence of rSurvivin (2 μM) was significantly higher than that in the absence of the protein as well as in the presence of HSA (2 μM) (Figure 4G). It is unclear why SSFP5 showed a higher increase in fluorescence intensity in the presence of survivin compared to other SSFPs with polyproline linkers. However, unlike SSFP3 and SSFP4, which each have only one polyproline linker, SSFP5 features two polyproline linkers near the center of the molecule. This configuration may result in an increased distance between the FAM and DABCYL moieties.

**Figure 5:**
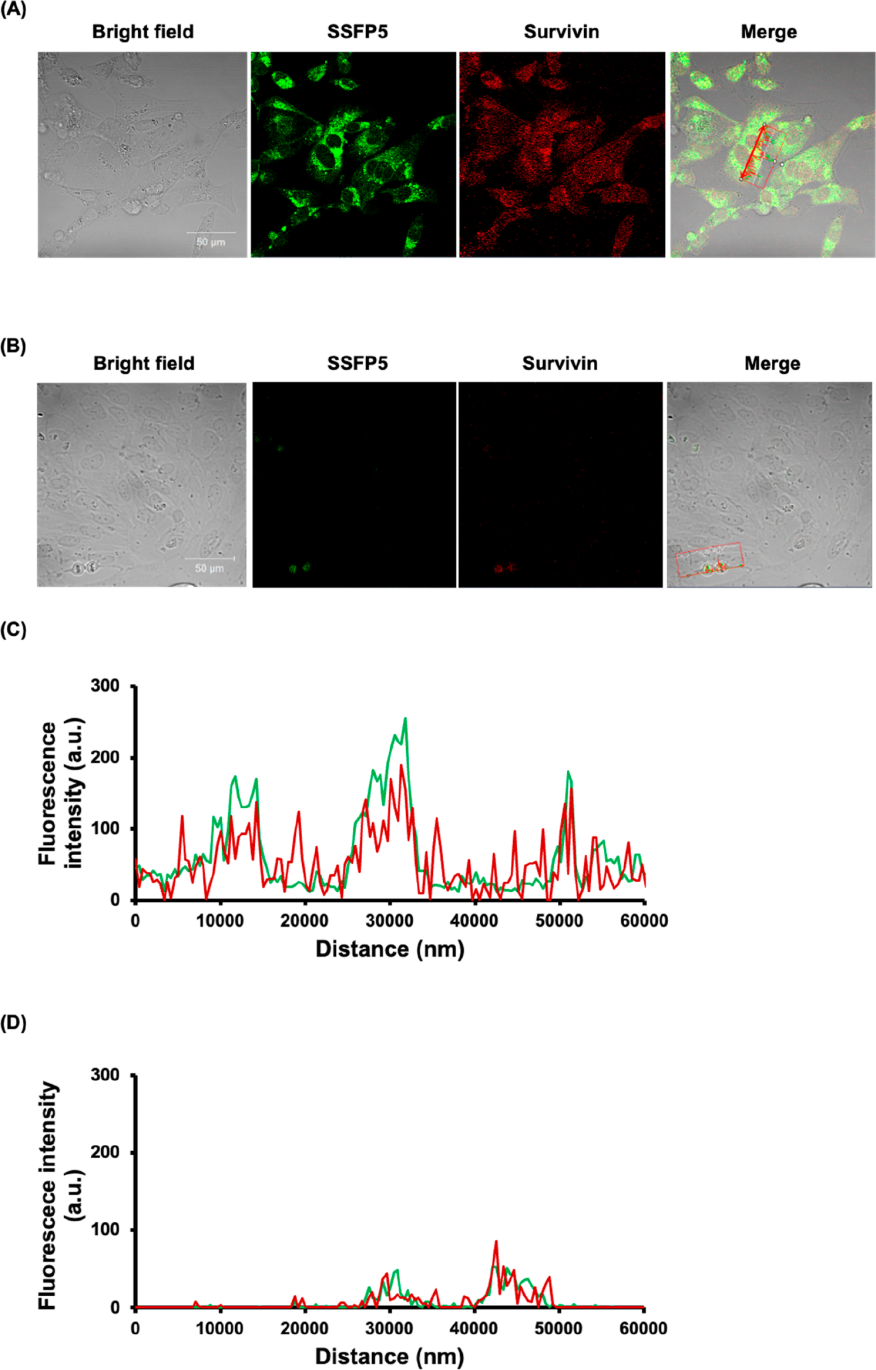
Representative confocal fluorescence microscopy images of SSFP5 (5 μM) in MDA-MB-231 (A) and MCF-10A (B) cells. Cells labeled with SSFP5 are shown in green, survivin protein stained with monoclonal antibody D-8 (Alexa Fluor 633) is shown in red. Merged images show colocalization of SSFP5 accumulation and survivin. Line analysis of fluorescence images of SSFP5 and survivin protein expression levels in MDA-MB-231 (C) and MCF-10A (D) cells (spectral marks in A and B, respectively). Green line and red line represent SSFP5 signals and survivin protein level, respectively.

Finally, we investigated whether SSFP5 showed differences in binding between cell lines with different levels of survivin expression. To address this, we conducted fluorescent double-staining experiments utilizing SSFP5 and an antisurvivin antibody in MDA-MB-231 cells, which have high survivin expression, and MCF-10A cells, which have low survivin expression.^25^ Membrane-permeabilized MDA-MB-231 and MCF-10A cells were exposed to 5 μM SSFP5 followed by fluorescent staining with a monoclonal antibody against survivin. Confocal microscopy revealed significant SSFP5 signals in the survivin-positive regions of MDA-MB-231 cells (Figure 5A). In contrast, minimal SSFP5 fluorescence signals were observed in the MCF-10A cells (Figure 5B). Additionally, quantitative line analysis indicated a strong correlation between SSFP5 accumulation in MDA-MB-231 cells and survivin expression (Figure 5C). Additionally, a partial correlation was observed between SSFP5 accumulation and survivin expression in MCF-10A cells; however, the signals were much weaker than those observed in MDA-MB-231 cells (Figure 5D). These findings suggest that SSFP5 effectively recognized cellular survivin. These SSFPs can be applied to screen novel survivin-binding agents and/or endogenous biomolecules with affinity for the binding sites of survivin and Borealin. Unlike immunohistochemistry, which requires complex procedures and expensive reagents including antibodies, SSFPs are anticipated to offer a more cost-effective and straightforward method for survivin detection in vitro. It should be noted that the SSFPs developed in this study are reagents intended for use in vitro rather than in vivo at the current stage. To utilize them as in vivo imaging probes, it appears imperative to enhance metabolic stability through the conversion from linear peptides composed of l-amino acids to peptides incorporating special amino acids or cyclic peptides. Fluorescence staining evaluations of cancer cells in this study were conducted using conditions employing SSFP5 at 5 μM (Figure 5), where clear fluorescence images were obtained, without considering toxicity. However, successful development of novel compounds for potential in vivo use in the future may necessitate evaluations at lower concentrations. Nonetheless, evaluations using lower concentrations of SSFP5 were also performed, but clear fluorescence images were not obtained (data not shown). The cause of this might be attributed to the ratio of fluorescence intensity between the presence and absence of survivin, which is approximately 2.66. Therefore, it may be necessary to develop SSFPs with higher contrast for potential applications. Recently, enzyme-activatable fluorescence probes were used for tumor diagnosis and image-guided surgery.^26^ Thus, survivin protein-specific turn-on type fluorescent probes may also serve as adjunctive agents in cancer surgery. The long peptide with residues 42–55 developed in this study is not highly membrane-permeable; therefore, additional membrane-permeable peptides are necessary to visualize survivin in living cells. Recently, membrane-permeable peptides, such as cR_10_, have been developed to deliver proteins larger than polypeptides, such as variable fragments of heavy chain antibodies, into cells.^27^ Therefore, it may be possible to develop fluorescent probes that can specifically capture survivin in vivo by fusing them with the SSFPs developed in this study. Additionally, SSFPs may be applied to survivin-targeting activatable photodynamic cancer therapy using appropriate FRET-activatable probes with a photosensitizer.^28^ This study is a pilot investigation into the development of linear peptides that may encounter challenges related to metabolic stability. Therefore, toxicity and stability assessments of these peptides were not conducted, as they are intended for use only in in vitro experiments. However, comprehensive studies on toxicity and metabolic stability are essential to substantiate the utility of improved SSFP derivatives as in vivo imaging agents in next studies. Anticipated advancements in this research field are imminent.

## Conclusions

In this study, we designed and synthesized SSFPs that can specifically capture survivin and evaluated them as survivin-specific FRET-activatable probes to elucidate the physiological functions of survivin in cancer and apply them to cancer diagnosis. All SSFPs exhibited an increased fluorescence intensity in the presence of rSurvivin. SSFP5, which features an extended linker and polyproline, showed the highest increase in fluorescence intensity. SSFP5 consistently exhibited fluorescence intensity that correlated with survivin expression in the fluorescence imaging of cell lines. These results demonstrated that SSFP5 specifically binds to survivin, leading to fluorescence emission. Although enhancements such as increased affinity and intensity are necessary, SSFP5 effectively functions as a survivin-sensitive fluorescent probe.

## Supporting Information Available

MALDI-TOF MS data and FRET quenching efficiency of SSFPs (PDF)
