# Supplementary material for: Peptide-Based Turn-On Fluorescent Probes for Highly Specific Detection of Survivin Protein in the Cancer Cells
Source: Chem Biomed Imaging. 2024 May 2;2(5):374–83. doi: 10.1021/cbmi.4c00017 (PMC11504145; doi:10.1021/cbmi.4c00017)
Supplement: Supplementary file 1 — im4c00017_si_001.pdf [file im4c00017_si_001.pdf]

Supporting Information

Peptide-based turn-on fluorescent probes for highly specific detection of survivin protein in the cancer cells

Takeshi Fuchigami[a*], Tomoe Nakayama[b], Yusuke Miyanari[c], Iori Nozaki[a,b], Natsumi Ishikawa[b], Ayako Tagawa[c], Sakura Yoshida[b], Masayuki Munekane[a], Morio Nakayama[b], Kazuma Ogawa[a,d]

[a] Laboratory of Clinical Analytical Sciences, Graduate School of Medical Sciences, Kanazawa University, Kakuma-machi, Kanazawa, Ishikawa 920-1192, Japan

[b] Department of Hygienic Chemistry, Graduate School of Biomedical Sciences, Nagasaki University, 1-14 Bunkyo-machi, Nagasaki 852-8521, Japan

[c] Institute of Nano Life Science, Kanazawa University, Ishikawa 920-1192, Japan

[d] Institute for Frontier Science Initiative, Kanazawa University, Kakuma-machi, Kanazawa, Ishikawa 920-1192, Japan;

Corresponding Author

Takeshi Fuchigami − Laboratory of Clinical Analytical Sciences, Division of Pharmaceutical Sciences, Kanazawa University Graduate School, Kakuma-machi, Kanazawa, Ishikawa 920-1192, Japan;

orcid.org/0000-0001-8141-1212; Email: t-fuchi@p.kanazawa-u.ac.jp*

Table of Contents

Table S1 .... S3

Figure S1 .... S4

Table S2 .... S6

Table S1. MALDI-TOF MS data of survivin-sensitive fluorescent probes (SSFPs)

SSFPs	Calculated MS $(M+H)^+$	Observed MS $(M+H)^+$
SSFP1	5115.4	5121.1
SSFP2	5918.4	5921.8
SSFP3	6404.0	6405.5
SSFP4	6057.9	6055.5
SSFP5	5962.3	5961.9

(A)

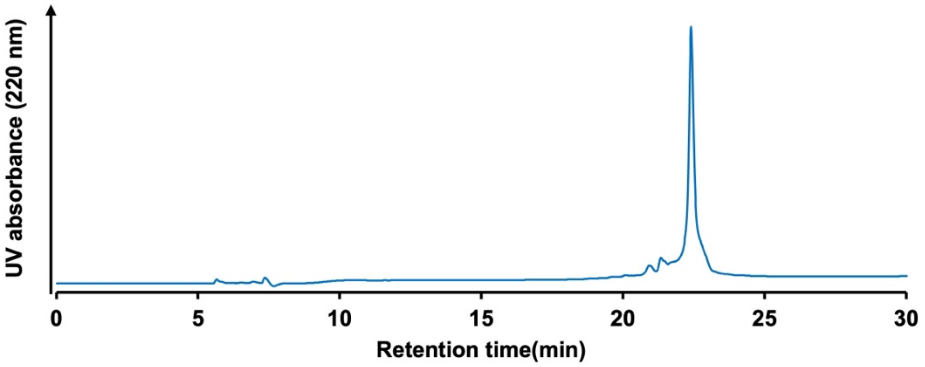
UV absorbance (220 nm)
0
5
10
15
20
25
30
Retention time(min)

(B)

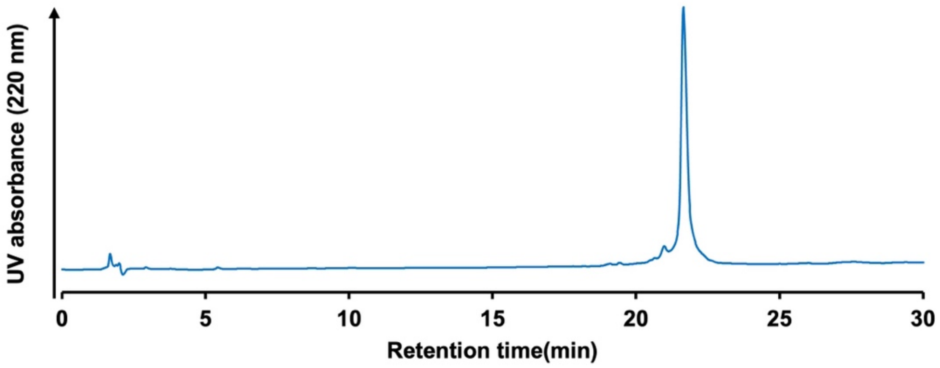
UV absorbance (220 nm)
0
5
10
15
20
25
30
Retention time(min)

(C)

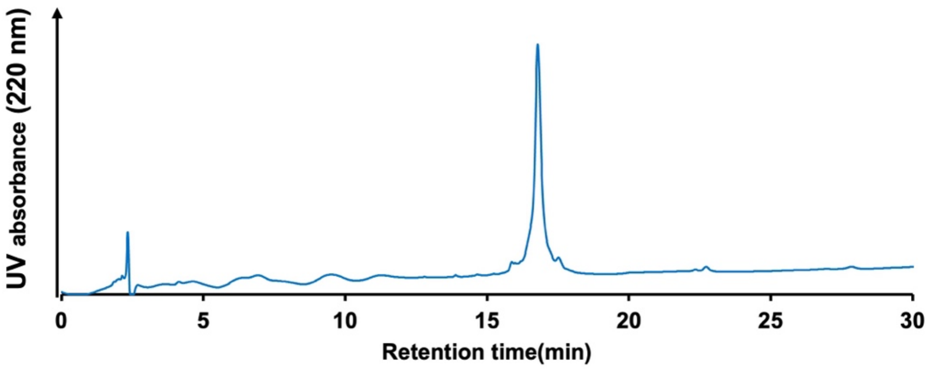
UV absorbance (220 nm)
0
5
10
15
20
25
30
Retention time(min)

(D)

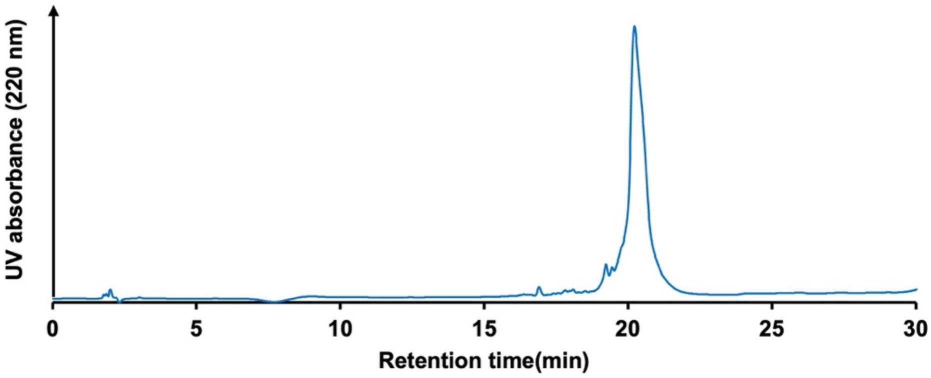

(E)

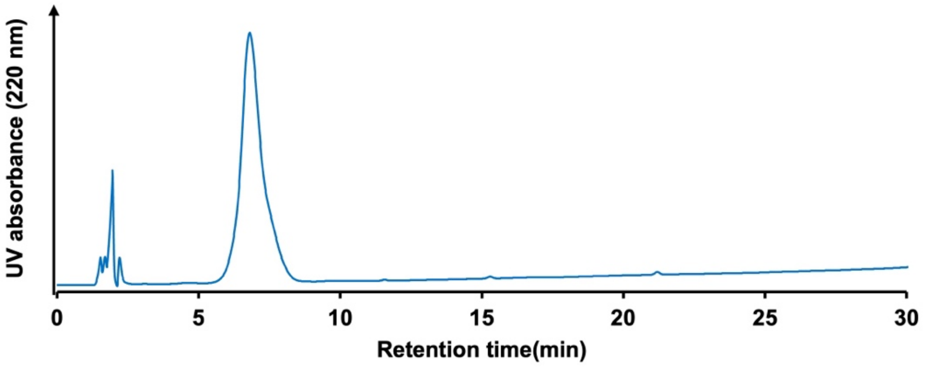

Figure S1. Analytical HPLC of purified SSFP1 (A), SSFP2 (B), SSFP3 (C), SSFP4 (D), and SSFP5 (E) using gradient elution of acetonitrile with 0.1% TFA/water with 0.1% TFA (B). Gradient time: 0–30 min. Gradient conditions: 20/80–80/20 (A), 30/70–70/30 (B), 40/60–60/40 (C), 30/70–70/30 (D), and 40/60–70/30 (E). Flow rate: 1 mL/min. Peaks within 2.5 min are from the solvent used to dissolve the sample.

Table S2. FRET quenching efficiency of SSFPs

SSFPs	FRET efficiency (%)*
SSFP1	99.6
SSFP2	98.2
SSFP3	95.7
SSFP4	95.7
SSFP5	98.3

*Measurement condition; ex: 483 nm, em: 525 nm.
